# Breaking the Linear Scaling Relations for the Oxygen Reduction Reaction with a Dual‐Atom Catalyst Composed of a MnFe‐Porphyrrole Aerogel

**DOI:** 10.1002/anie.202514013

**Published:** 2025-09-13

**Authors:** Eliana Lebowitz, Prasenjit Das, Łukasz Kielesiński, Leigh Peles‐Strahl, David A. Cullen, Ilya Grinberg, Daniel T. Gryko, Lior Elbaz

**Affiliations:** ^1^ Chemistry Department, Bar‐Ilan Center for Nanotechnology and Advanced Materials Bar‐Ilan University Ramat‐Gan 5290002 Israel; ^2^ Institute of Organic Chemistry of Polish Academy of Sciences Warsaw 01–224 Poland; ^3^ Center for Nanophase Materials Sciences Oak Ridge National Laboratory Oak Ridge TN 37831 USA

**Keywords:** Aerogel, Corrole, Oxygen reduction Reaction, PGM‐free, Porphyrin, Scaling relations

## Abstract

Bimetallic catalysts offer enhanced catalytic performance through synergistic interactions between the two metals, allowing them to break the linear scaling relations and reach high electrocatalytic activity. This study presents bimetallic aerogel‐based catalyst synthesized as a covalent, three‐dimensional framework containing neighboring iron and manganese sites. The aerogel structure provides a high surface area and porosity, facilitating an ultra‐high active site density and efficient mass transport. The MnFe porphyrrole's unique structure is obtained by alternately linking Mn‐porphyrin and Fe‐corrole complexes. It exhibited outstanding performance with an onset potential of 0.99 V_RHE_. Comparative studies with a free‐base Fe porphyrrole catalyst (*E*_onset_ 0.97 V_RHE_) revealed that while Mn incorporation led to only a slight improvement in half‐cell performance, it resulted in significantly enhanced performance in anion exchange membrane fuel cell. The MnFe catalyst achieved an OCV of 0.97 V and a peak power density of 0.27 W cm^−^^2^, outperforming the free‐base Fe counterpart. Using density functional theory calculations, we show that the higher ORR activity of MnFe‐porphyrrole is due to charge transfer between Mn and Fe atoms, which is absent in the reference free‐base Fe‐porphyrrole. These findings underscore the advantages of bimetallic catalysts in improving ORR activity and fuel cell efficiency by leveraging synergistic effects.

## Graphical abstract

A bioinspired MnFe porphyrrole aerogel catalyst is synthesized using a covalent 3D framework with adjacent metal centers. The bimetallic structure enhances ORR activity through Mn─Fe synergy and high active site density. Fuel cell tests show over a 30% increase in peak power density, with DFT calculations confirming metal–metal charge transfer as the source of improved performance.

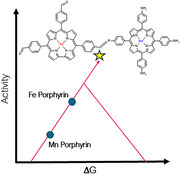

## Introduction

The oxygen reduction reaction (ORR) is a critical process in energy conversion, particularly in fuel cell technologies. However, its sluggish kinetics present a significant challenge that must be addressed to make fuel cells viable for widespread use.^[^ ^1^, ^2^ ^]^ Traditionally, platinum‐group metal (PGM) catalysts have been employed to facilitate this reaction due to their superior activity,^[^ ^3^ ^]^ but their high cost and limited natural abundance have spurred extensive research aimed at developing more sustainable alternatives. Recently, there has been a concerted effort to explore catalysts that are both cost‐effective and free of critical raw materials, focusing on PGM‐free compounds.^[^ ^4^ ^]^

In response to these challenges, substantial research efforts have been directed toward developing PGM‐free catalysts that are more cost‐effective and economically viable, yet capable of matching or even surpassing the efficiency of PGM‐based catalysts.^[^ ^5^ ^]^ One strategy to address this is to use PGM‐free materials and increase the electrochemically active site density by increasing the loading and surface area of the catalyst.^[^ ^5^, ^6^, ^7^, ^8^, ^9^ ^]^ By maximizing the surface area, even metals that are inherently less active than platinum can achieve high overall catalytic activity, potentially rivaling that of platinum.^[^ ^5^ ^]^ By incorporating these metals into materials like aerogels—highly porous structures with extensive active sites, surface area, and pore volume—it is possible to enhance the activity of PGM‐free ORR catalysts, compensating for their intrinsically low turnover frequency relative to platinum.^[^ ^5^, ^10^ ^]^

Another approach to improving ORR catalysts is biomimetics: drawing inspiration from nature's highly efficient enzymes.^[^ ^11^ ^]^ For this research, the active site in cytochrome c oxidase (CcO) was selected as a model due to its very efficient electrocatalysis of water at a relatively low overpotential.^[^ ^12^ ^]^ CcO's active site is bimetallic, consisting of a single iron atom in close proximity to a single copper atom. Despite its remarkable enzymatic activity, however, CcO is unsuitable for use in fuel cells due to its bulkiness, chemical sensitivity, and poor electrical conductivity.^[^ ^13^, ^14^ ^]^ Therefore, the catalyst synthesis focused on replicating only the active center of CcO to reduce bulkiness and enhance practicality for fuel cell applications.

CcO served as the primary inspiration for this work; however, other enzyme‐based systems, including peroxidase‐inspired Fe─N─C catalysts, have also been developed for ORR. Peroxidases catalyze hydrogen peroxide reduction rather than molecular oxygen activation, but the structural similarity of their heme environments to those of oxidases has led to the development of peroxidase‐inspired Fe─N─C catalysts with notable ORR activity.^[^ ^5^, ^11^, ^15^, ^16^, ^17^, ^18^, ^19^, ^20^, ^21^, ^22^ ^]^ These parallel approaches underscore the broader utility of heme‐based motifs, while the bimetallic nature of CcO continues to offer a unique framework for exploring cooperative metal‐site effects in O_2_ activation.

In addition to enabling enzymatic mimicry, bimetallic systems offer a promising strategy for overcoming fundamental catalytic limitations, such as the linear scaling relationship that governs the binding energies of ORR intermediates. In conventional single‐metal catalysts, improvements to one step in the reaction pathway come at the expense of others due to correlated energetics between intermediates like *OOH, *O, and *OH.^[^ ^23^ ^]^ This tradeoff imposes a theoretical ceiling on catalytic performance. However, placing two electronically distinct metal centers in close proximity, as observed in CcO, can decouple these relationships, enabling synergistic interactions that selectively stabilize specific intermediates and thereby break the linear scaling constraint.

In this work, a covalent organic framework (COF) aerogel was synthesized through alternating covalent binding of manganese porphyrin and iron corrole, forming an MnFe porphyrrole. The synthesis was designed to ensure that the reaction occurred exclusively between the porphyrin and corrole, preventing the coupling of identical units. This strategy positioned two distinct metal centers in close proximity, thus mimicking the bimetallic active site of CcO, with the exception that manganese was used instead of copper. The aerogel was then subjected to pyrolysis to improve its electronic conductivity. To evaluate the effect of bimetallic interactions, a second aerogel was synthesized using the same method, replacing the manganese porphyrin with a free‐base porphyrin to create a single‐metal reference catalyst. Comprehensive characterization was conducted to verify the success of both syntheses and to assess their catalytic properties through electrochemical measurements.

This templated synthesis approach, starting from a well‐defined COF, provides a controlled pathway for developing new catalysts that have the potential to break linear scaling relations and achieve exceptional catalytic activity.^[^ ^24^ ^]^ Pioneering work by Dey et al. has demonstrated that tuning the ligand environment in iron porphyrins, particularly through strategies inspired by the distal hydrogen‐bonding network of horseradish peroxidase (HRP), can effectively break linear scaling relations and enhance ORR performance.^[^ ^16^, ^18^, ^19^, ^20^, ^21^, ^22^ ^]^ Building on these important insights, this work uses a complementary strategy based on the incorporation of a neighboring manganese site to induce synergistic bimetallic effects. The resulting porphyrrole structure offers a platform for exploring synergistic interactions between adjacent metal centers, enabling oxygen activation beyond conventional single‐site limitations.

## Results and Discussion

Herein, Fe‐corrole was covalently bonded to Mn‐porphyrin using a Schiff base reaction between the amino (NH_2_) groups on the Mn‐ porphyrin and the aldehyde (CHO) groups on the Fe‐corrole. The synthesis strategy aimed to construct a framework in which porphyrins are exclusively bonded to corroles, and corroles are bonded solely to porphyrins, thereby avoiding any porphyrin–porphyrin or corrole–corrole linkages. This selective bonding was feasible due to the distinct functional groups on the porphyrins and corroles.^[^ ^25^, ^26^, ^27^, ^28^, ^29^ ^]^ While the Mn‐porphyrin precursor is commercially available, a multi‐step synthesis is required to obtain the Fe‐corrole precursor. First, the suitable A_3_‐corrole (**1**) with formyl groups was prepared as was recently described by us.^[^ ^25^ ^]^ This compound (**1**) was then used to synthesize the iron corrole complex using anhydrous FeCl_2_, as performed in the literature.^[^ ^26^, ^27^, ^28^ ^]^ The reaction was conducted in DMF and heated at 120 °C for 1 hour under an argon atmosphere (Scheme S1). In the preparation of the Fe‐corrole product (**2**), *μ*‐oxodiiron(IV) corrole complex was also formed as a byproduct, which was confirmed by nuclear magnetic resonance (NMR) spectroscopy (signals in the range from 6.6 to 8.1 ppm) and mass spectrometry (MS) analysis.^[^ ^29^ ^]^ Therefore, we decided to transform iron(IV) *μ*‐oxo dimer to the compound (**2)**. Crude precipitate was dissolved in DCM and stirred with 7% solution of HCl for two days at room temperature. This procedure afforded the pure iron complex (**2**) with 79% yield. In the polymerization reaction of Mn‐porphyrin and Fe‐corrole, the desired C═N bonds formed between the two complexes, resulting in a MnFe‐porphyrrole gel.

**Figure 1:**
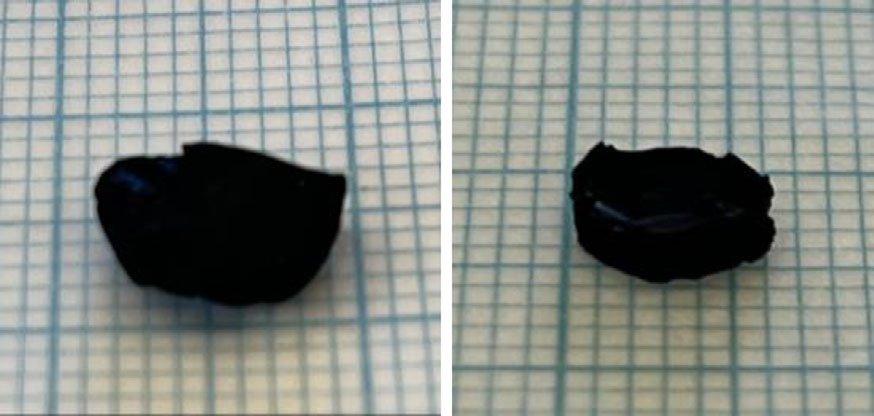
Left, MnFe aerogel before pyrolysis; right, MnFe aerogel after pyrolysis (HT‐MnFe).

The resulting gel was dried using a supercritical dryer and subsequently pyrolyzed at 800 °C to enhance its electrical conductivity to give a HT‐MnFe‐porphyrrole aerogel. The pyrolysis temperature of 800 °C was selected based on previous work with similar aerogels.^[^ ^5^ ^]^ Thermogravimetric analysis (TGA) indicated a 31% mass loss at this temperature, consistent with an earlier study that reported comparable results and identified 800 °C as optimal for catalytic activity,^[^ ^6^ ^]^ confirming the suitability of the chosen conditions. Figure 1 shows the aerogel before and after pyrolysis. Following pyrolysis, the MnFe aerogel (HT‐MnFe) exhibits a reduction in volume while retaining its black coloration.

**Figure 2:**
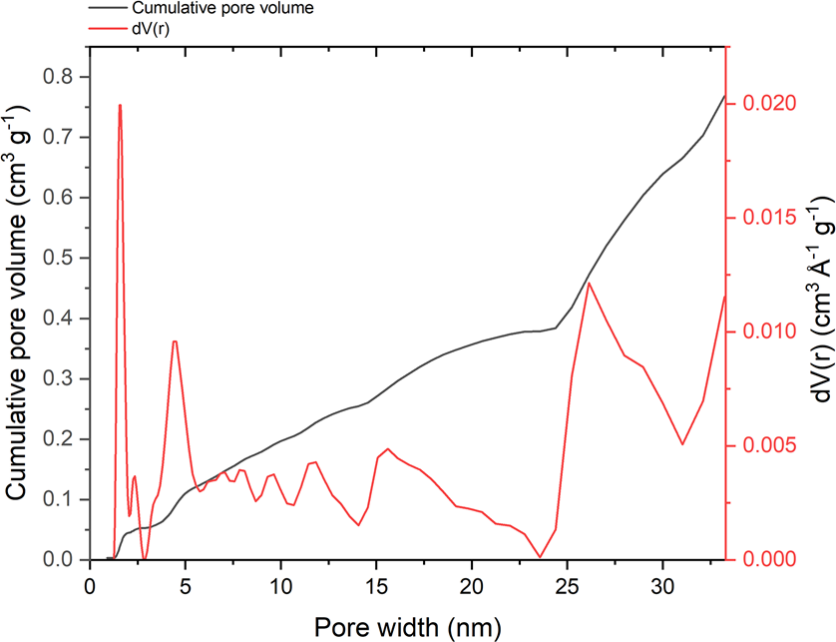
DFT pore size distribution and cumulative pore volume.

To assess the specific surface area and pore size distribution, BET measurements were conducted. Nitrogen adsorption isotherms were recorded, and the surface area was calculated using the BET model. The pore size distribution was determined through density functional theory (DFT) analysis (Figure 2). The resulting BET surface area of 342 m^2^g^−1^ confirms the successful synthesis of the aerogel and indicates the potential for a high electrochemically active site density and effective catalytic performance. The pore size distribution shows that there is a variety of pore sizes in the aerogel, forming a hierarchical structure with micropores accounting for 6% of the total pore volume and mesopores comprising the remaining 94%. Notably, 10% of the mesopores are smaller than 5 nm. This hierarchical arrangement is known to assist with mass transport and overall electrochemical efficiency.^[^ ^30^, ^31^ ^]^

Inductively coupled plasma measurement (ICP) analysis was used to examine the Fe and Mn loadings, with an expected atomic ratio of 1:1.33 (Mn:Fe) based on the synthesis method. The actual measured ratio was 1.0:1.4, slightly favoring Fe, which is close to the anticipated 1:1.33 balance. The deviation from the expected ratio may result from variations in the coordination strength of Mn─N and Fe─N bonds.^[^ ^32^, ^33^ ^]^ Nevertheless, the relative amounts of both metals suggest the successful formation of the intended bimetallic catalytic structure.

**Figure 3:**
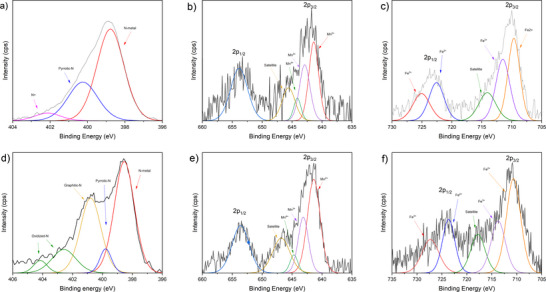
Top row: XPS before pyrolysis a) Nitrogen, b) Manganese, and c) Iron. Bottom row: XPS after pyrolysis d) Nitrogen, e) Manganese, and f) Iron.

The metal‐ligand interactions within the aerogel were studied using X‐ray photoelectron spectroscopy (XPS) (Figure 3). Prior to heat treatment, the Mn 2p spectrum displayed peaks at 642.88 and 654.38 eV, corresponding to the 2p_3/2_ and 2p_1/2_ spin components, respectively. Deconvolution revealed a mixture of Mn^2^⁺, Mn^3^⁺, and Mn⁴⁺ species, with no signal near 638 eV, the characteristic binding energy of metallic Mn. After pyrolysis, the overall spectral features remained nearly identical. The 2p_3/2_ and 2p_1/2_ peaks shifted slightly (∼0.2 eV) to lower binding energies, which is within the expected range of instrumental variation and consistent with subtle changes in the surrounding carbon matrix, such as increased graphitization. The distribution of oxidation states also remained largely the same, with Mn^2^⁺ continuing to dominate and minor contributions from Mn^3^⁺ and Mn⁴⁺ still present. Metallic Mn was not observed after pyrolysis, further supporting that the Mn center remains in an oxidized, coordinated state. These results indicate that the pyrolysis process does not significantly alter the Mn coordination environment.^[^ ^34^ ^]^

The Fe 2p XPS spectra before and after pyrolysis show that the iron centers remain in oxidized states, with no evidence of metallic Fe. Before heat treatment, peaks were observed at 709.68 and 711.48 eV, corresponding to the 2p_3/2_ components of Fe^2^⁺ and Fe^3^⁺, respectively, along with corresponding 2p_1/2_ peaks at 722.68 and 725.08 eV. A satellite feature was present at 717.63 eV, typical of Fe in mixed oxidation states. After pyrolysis, these features remain nearly unchanged in position, with no signal near 706.8 eV, confirming the absence of metallic Fe. A slight increase in the intensity of the Fe^2^⁺ component was observed after pyrolysis, indicating a modest shift in the Fe^2^⁺/Fe^3^⁺ ratio, possibly due to changes in the electronic environment or coordination with nitrogen species in the carbonized matrix. The satellite shift to lower binding energy is consistent with the increase in Fe^2+^ character.^[^ ^34^, ^35^ ^]^ Nevertheless, the overall similarity in peak structure and the persistent absence of metallic features suggest that the iron coordination environment is largely retained following pyrolysis.

The N 1s spectrum of the MnFe porphyrrole aerogel before pyrolysis is composed of three peaks. The first peak, at 398.78 eV, corresponds to the N‐metal bond. The second peak, at 400.28 eV, is attributed to pyrrolic‐N, while the third peak, at 402.18 eV, is associated with charged‐N, which arises from the imine‐like bond formed between the porphyrin and the corrole during polymerization. After pyrolysis, these peaks shifted slightly to lower binding energies: 398.42 eV for N‐metal and 399.78 eV for pyrrolic‐N. Additionally, a new peak appeared at 400.78 eV, which is attributed to graphitic‐N. Two more peaks at higher binding energies were also observed, corresponding to oxidized‐N.^[^ ^25^ ^]^ Overall, the XPS measurements indicate that the pyrolysis primarily affected the surrounding carbon atoms, making the carbon matrix around the center more graphitic and altering the ratio between pyrrolic‐N and graphitic‐N. However, the coordination environment of Mn and Fe remained largely unchanged compared to the aerogel before pyrolysis, as demonstrated by the N‐metal making up approximately 85% of the nitrogen bonds both before and after pyrolysis.

**Figure 4:**
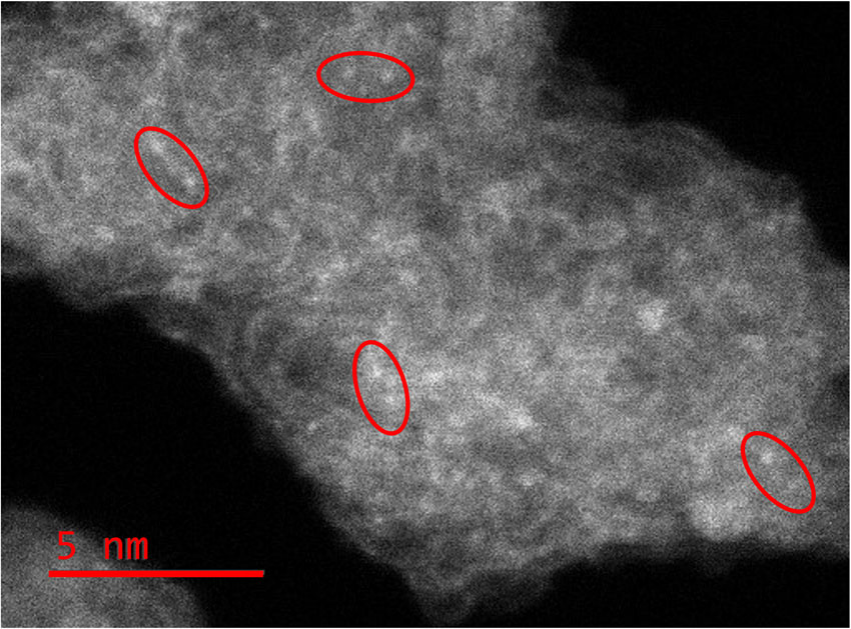
HR‐TEM image of heat‐treated MnFe porphyrrole, with examples of pairs of individual atoms, presumably iron and manganese, outlined in red.

To complement the XPS analysis, high‐resolution transmission electron microscopy (HR‐TEM) was performed, revealing the graphitization of the carbon matrix following pyrolysis, depicted in the ribbon‐like structure (Figure 4). The HR‐TEM images also show the presence of heavy atoms integrated within the aerogel structure appearing in pairs, which is attributed to Mn and Fe. The metal sites are atomically dispersed throughout the pyrolyzed aerogel.

**Figure 5:**
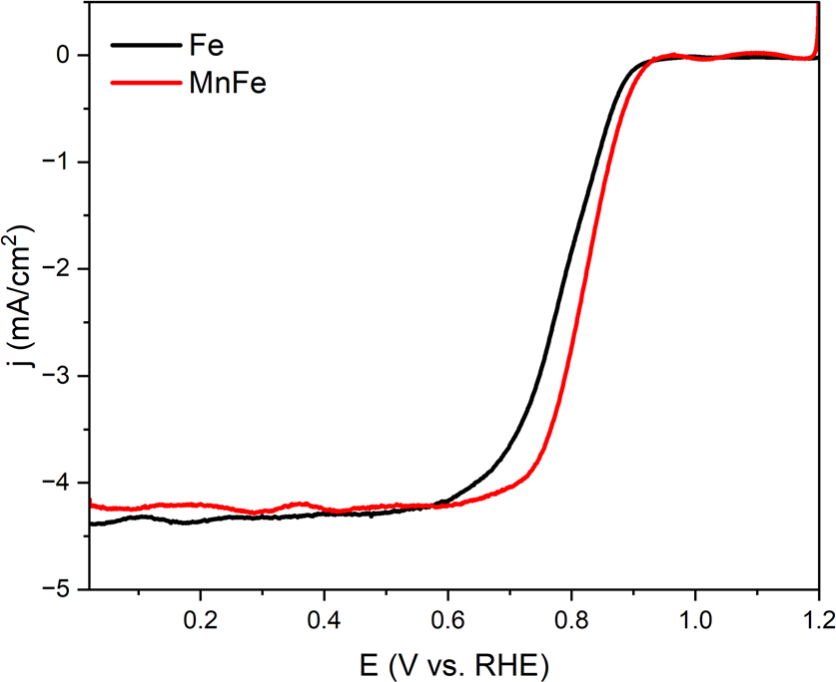
RDE results for MnFe and Fe porphyrroles.

**Figure 6:**
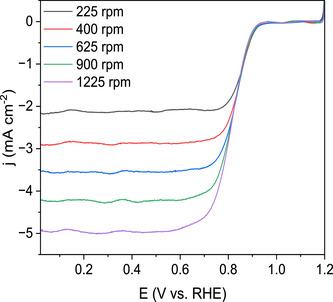
RDE results for MnFe porphyrrole at different rotation speeds.

Electrochemical analysis was performed using a rotating ring‐disk electrode (RRDE) in 0.1 M of KOH in deionized water. The results of the heat‐treated bimetallic MnFe porphyrrole demonstrated superior catalytic activity to that of the monometallic Fe porphyrrole (FBFe) (Figures 5 and 6). The onset potential (*E*_onset_) is 0.99 V versus RHE, and the half‐wave potential (*E*_1/2_) is 0.82 V versus RHE. The limiting current indicates a four‐electron reduction of O_2_ to OH^−^, as determined using the Levich Equation:

$$J=0.201nFD^{2/3}\upsilon^{-1/6}\omega^{1/2}C$$

where n represents the number of electrons transferred, *F* is the Faraday constant (96485 C mol^−1^), *D* is the diffusion coefficient in 0.1 M KOH in deionized water (1.9 x 10^−5^), *C* is the bulk oxygen concentration (1.15 x 10^−6^ mol mL^−1^), *ν* is the kinematic viscosity (0.01 cm^2^ s^−1^), and ω denotes the rotation speed (rpm). The number of electrons transferred in the reaction, determined using various rotation speeds, is *n* = 4.

The RRDE ring electrode was held at 1.2 V versus RHE to monitor the formation of peroxide anions during ORR. Throughout the entire potential range scan on the disk electrode, the current detected was minimal. The highest yield of HO_2_^−^ was observed at 0.171 V versus RHE, reaching 3.57%, as calculated using the following Equation:

$${\;\mathrm{HO}}_{2}^{-\;}=\frac{100\;x\;\frac{2I_{R}}{N}}{I_{D}+\frac{I_{R}}{N}}$$

where, I_D_ represents the disk current, I_R_ denotes the ring current, and N is the collection efficiency of the ring electrode.

This finding further supports the conclusion that the HT‐MnFe porphyrrole aerogel facilitates a four‐electron ORR pathway.

To quantitatively assess the activity of the HT‐MnFe porphyrrole catalyst, the kinetic current density (*j*_k_) was measured at 0.80 V versus RHE using the Koutecký–Levich equation, based on RRDE measurements taken at 225, 400, 625, 900, and 1225 rpm. A *j*_k_ value of 8.03 mA cm^−2^ was obtained, indicating strong intrinsic catalytic activity in alkaline media (Figure S2). This substantially exceeds the 2.22 mA cm^−2^ reported for a Fe─N─C catalyst, also derived from porphyrin, tested in 0.1 M KOH.^[^ ^36^ ^]^ The higher j_k_ observed for the HT‐MnFe porphyrrole suggests enhanced active site efficiency, likely arising from the synergistic bimetallic configuration and the tailored porphyrin–corrole architecture.

**Figure 7:**
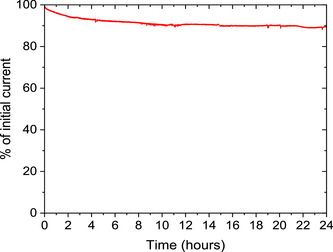
Durability test at 0.65 V vs. RHE, O_2_ saturated 0.1 M KOH at 900 rpm.

The durability of the HT‐MnFe porphyrrole aerogel was evaluated using chronoamperometry at 0.65 V versus RHE in 0.1 M KOH (Figure 7). The material exhibited a small initial decrease in current of ∼10% within the first 8 h, which may be attributed to the loss of less stable sites, such as edge‐bound metals or centers in lower oxidation states, which are generally more weakly coordinated to nitrogen ligands.^[^ ^37^ ^]^ After this initial drop, the current stabilized and remained essentially unchanged for the remainder of the 24 h test. No signs of catalyst failure were observed, indicating that the HT‐MnFe porphyrrole aerogel could likely sustain operation for longer periods of time. These results demonstrate that the HT‐MnFe porphyrrole aerogel possesses high intrinsic durability, highlighting its promise for long‐term applications.

**Figure 8:**
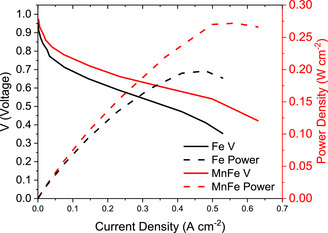
AEMFC performance of HT‐MnFe porphyrrole aerogel.

To confirm that the high performance observed for the HT‐MnFe porphyrrole aerogel using RRDE could be replicated in fuel cell conditions, a membrane electrode assembly was fabricated and tested in an anion exchange membrane fuel cell (AEMFC). The aerogel showed excellent performance in the AEMFC (Figure 8), achieving an open circuit voltage (OCV) of 0.97 V and a peak power density of 0.27 W cm^−2^. In comparison, the reference free base Fe porphyrrole exhibited significantly lower performance, despite having the same OCV of 0.97 V. The Fe porphyrrole showed a much steeper voltage drop in the kinetic region of the polarization curve and a lower peak power density of only 0.20 W cm^−2^, over 25% less than that of HT‐MnFe. Furthermore, the HT‐MnFe porphyrrole aerogel demonstrated a substantial improvement in the resistance‐limited region of the polarization curve (0.05–0.5 A cm^−2^), maintaining a more stable voltage and reducing overall losses. These preliminary results are highly encouraging, suggesting that Mn incorporation plays a critical role in enhancing fuel cell performance, and further optimization may lead to even greater improvements.

A density functional theory (DFT) study was performed for a model porphyrrole system as well as for the component Mn‐porphyrin and Fe‐corrole systems to determine the origin of the observed enhanced performance for MnFe‐porphyrrole in comparison to the reference free‐base Fe‐porphyrrole. The DFT study focused on the free energy of OH adsorption (Δ*G*_ads,OH_) relative to water on porphyrrole, porphyrin, and corrole catalysts because this parameter is an indicator of ORR activity within the framework of the computational hydrogen electrode (CHE) model introduced by Nørskov and co‐workers.^[^ ^38^ ^]^ Previous work showed that Δ*G*_ads,OH_ values within the range of 0.7 to 1.0 eV indicate high ORR activity and high OCV values. Since the extended polymerization framework and the presence of Mn and Fe atoms in closest proximity can influence the local bonding environment at the active site in the MnFe‐porphyrrole, we expect that the Δ*G*_ads,OH_ values on Fe and Mn sites of the MnFe‐porphyrrole will be different from the Δ*G*_ads,OH_ values of the individual component Mn‐porphyrin and Fe‐corrole systems.

**Figure 9:**
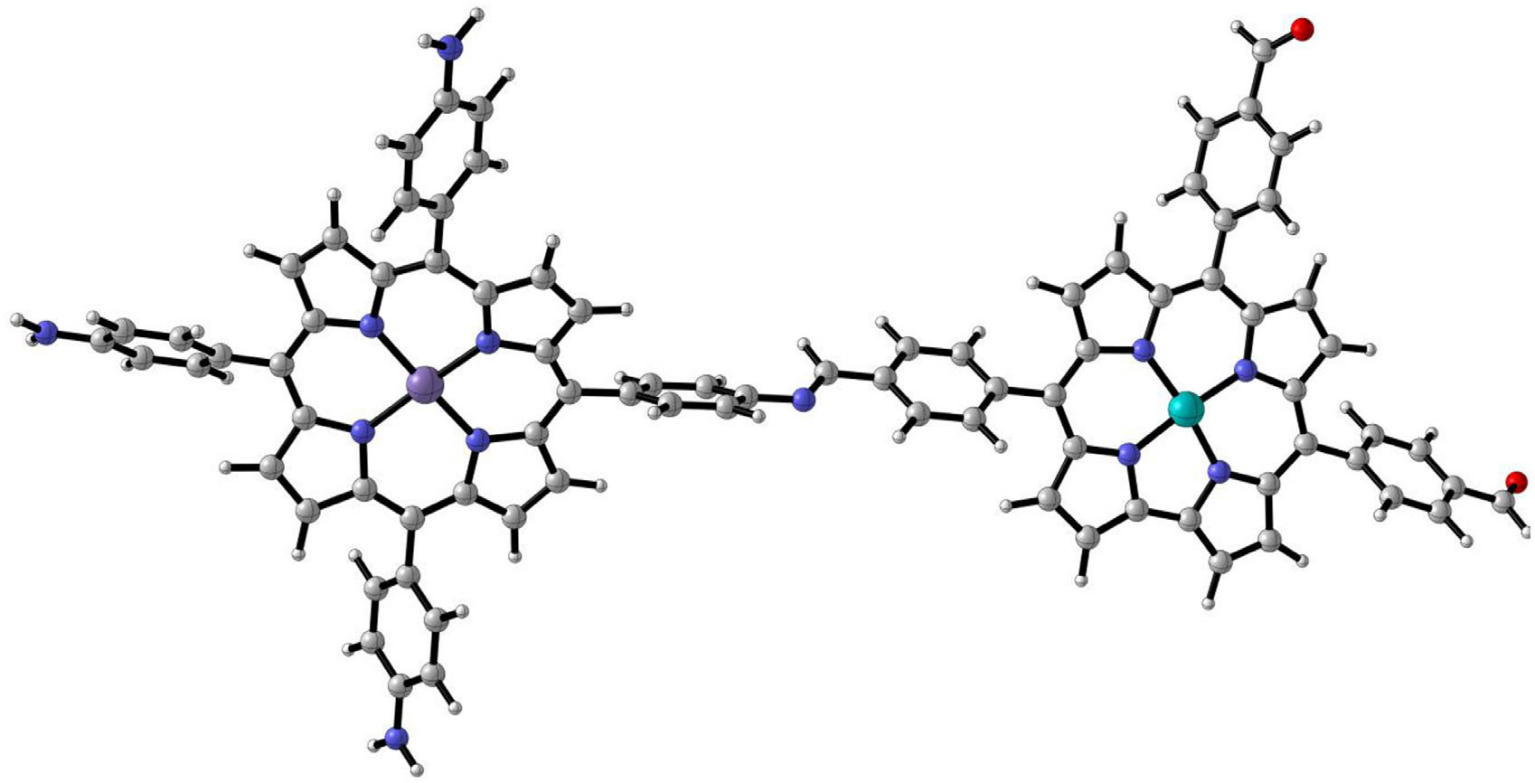
One‐dimensional MnFe‐porphyrrole structure considered in this study. White, gray, blue, red, cyan, and violet spheres represent H, C, N, O, Fe, and Mn atoms, respectively.

While the porphyrrole has a three‐dimensional configuration in the experimentally studied aerogel, a three‐dimensional porphyrrole system is too large and will incur too high a computational cost in our calculations. Therefore, we have considered a model MnFe‐porphyrrole system shown in Figure 9 that includes the effects of extended carbon networks and the closest proximity of Mn and Fe atoms, while still being computationally tractable.

Because it includes the effects of the proximity of the Mn and Fe atoms and the presence of an extended carbon network, this model system should provide information about the key difference in the active centers during the ORR catalysis due to the formation of the porphyrrole.

The computed Δ*G*_ads,OH_ values are presented in Table 1. For the Mn‐porphyrin and Fe‐corrole systems, the difference in Δ*G*_ads,OH_ values between the def2‐TZVP and def2‐TZVPP basis sets was found to be only 0.04 eV. As the size of the modeled Fe‐porphyrrole and MnFe‐porphyrrole systems is large, we have performed single‐point energy calculations for these systems only at the def2‐TZVP basis set, and a correction of 0.04 eV was applied to approximate the def2‐TZVPP level results. The Δ*G*_ads,OH_ values indicate that Mn‐porphyrin and Fe‐corrole are poor catalysts as they bind the reactive intermediates too strongly. Relative to the Δ*G*_ads,OH_ of the Fe‐corrole, the Δ*G*_ads,OH_ values at the Fe site increase by 0.26 and 0.43 eV to 0.70 and 0.86 eV in Fe‐porphyrrole and MnFe‐porphyrrole, respectively.

**Table 1:** Estimated OH adsorption free energies (Δ*G*_ads,OH_) of various active sites. The values without and within parentheses are obtained at the PBE/def2‐TZVP and PBE/def2‐TZVPP levels, respectively.

Complex	Mn‐porphyrin	Fe‐corrole	MnFe‐porphyrrole	MnFe‐porphyrrole	Fe‐porphyrrole
**Active site**	Mn	Fe	Mn	Fe	Fe
**Δ*G*_ads, OH_**	0.31 (0.35)	0.40 (0.44)	0.18 (0.22)	0.82 (0.86)	0.66 (0.70)

Interestingly, these values are within the required range of 0.7–1.0 eV, with the 0.86 eV obtained for the Fe site of the MnFe‐porphyrrole close to the top of the volcano curve for ORR. The MnFe‐porphyrrole shows a higher Δ*G*_ads,OH_ value by 0.16 eV at the Fe site than the Fe‐porphyrrole, indicating weaker binding of OH, which results in higher ORR catalytic activity, in agreement with the experimental results. In contrast to the Fe site, the calculated Δ*G*_ads,OH_ values show that the Mn site in the MnFe‐porphyrrole exhibits stronger binding with OH compared to the Mn‐porphyrin system.

We propose that the changes in the Δ*G*_ads,OH_ values of the Fe and Mn sites are due to charge transfer effects enabled by the porphyrrole structure. A lower charge of the Fe site is likely to lead to weaker binding of the electron‐rich OH and a higher Δ*G*_ads,OH_ value. Conversely, a higher charge of the Mn site is likely to lead to stronger binding of the electron‐rich OH and a lower Δ*G*_ads,OH_ value. In the free‐base Fe‐porphyrrole system, the added porphyrin acts as an electron‐donating group to the Fe site through the extended carbon networks, raising Δ*G*_ads,OH_. Then, the introduction of Mn into the porphyrin of the porphyrrole leads to the carbon‐network‐mediated charge transfer from Mn ion to the Fe ion due to the greater electronegativity of Fe, leading to a further increase in Δ*G*_ads,OH_. The loss of electrons by Mn to Fe increases the strength of OH binding on the Mn site, decreasing the Δ*G*_ads,OH_ at the Mn site of the MnFe‐porphyrrole relative to that of Mn‐porphyrin.

**Figure 10:**
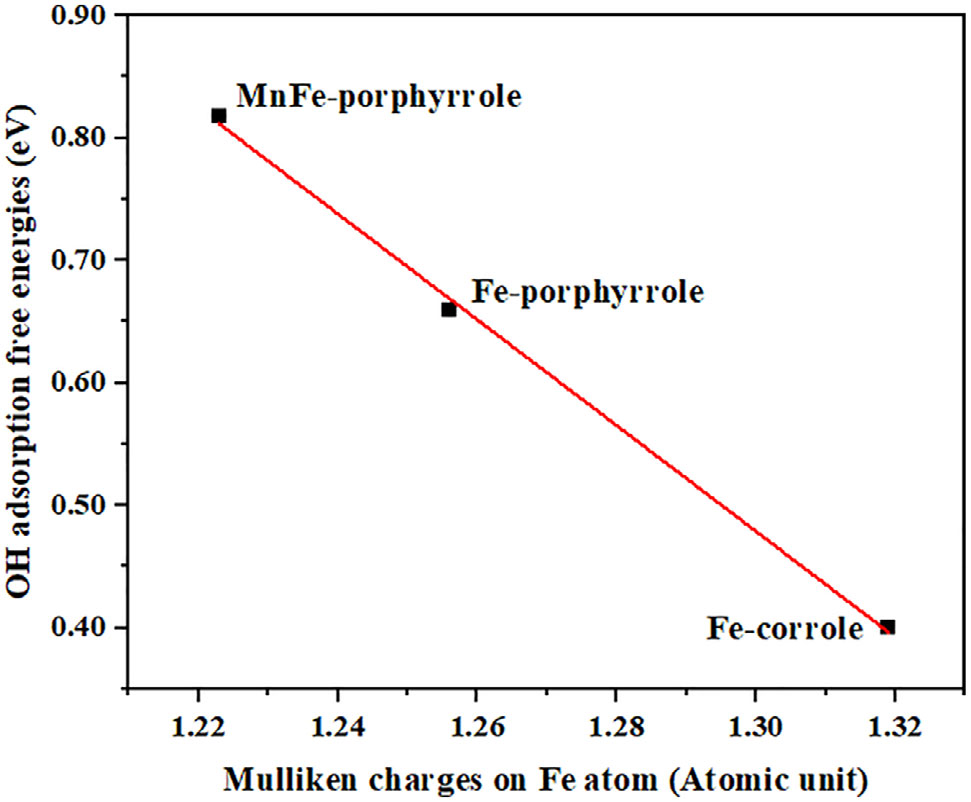
Correlation between the Mulliken charges on the Fe atom and the DFT‐calculated values of OH adsorption free energies (ΔG_ads,OH_).

To verify the above‐mentioned hypothesis, we computed the Mulliken charges^[^ ^39^ ^]^ on the Fe site in the Fe‐corrole, Fe‐porphyrrole, and MnFe‐porphyrrole systems, and the values are 1.319, 1.256, and 1.223 atomic units, respectively. The Mulliken charge on the Mn site (1.347 atomic units) is greater than that in the Fe site (1.223 atomic units) in MnFe‐porphyrrole. From these values, we can see that in the MnFe‐porphyrrole system, the charge on the Fe site is lowest, indicating that charge is transferred from the Mn site to the Fe site. While this charge transfer is absent in Fe‐porphyrrole, due to the presence of extended polymerization, the charge on the Fe site in Fe‐porphyrrole is lower than that of the Fe‐corrole. To quantify the relationship between ion charge and Δ*G*_ads,OH_, we plot the Δ*G*_ads,OH_ values for the Fe site versus the Fe Mulliken charges (Figure 10). This plot shows the expected negative correlation between the Δ*G*_ads,OH_ values and the charges, with an excellent linear relationship between the Fe charge and the OH adsorption free energy. Similar plots are obtained when the Δ*G*_ads,OH_ values are plotted versus the Hirshfeld,^[^ ^40^ ^]^ Löwdin,^[^ ^41^ ^]^ Voronoi Deformation Density (VDD),^[^ ^42^ ^]^ and CM5 charges^[^ ^43^ ^]^ on the Fe site for the complexes (Figure S3), indicating that this relationship is independent of the method for the calculation of atomic charge. Therefore, we confirmed that the charge transfer mechanism is responsible for the higher ORR activity of MnFe‐porphyrrole than free base Fe‐porphyrrole. Based on this analysis, the charge transfer mechanism demonstrated for the MnFe‐porphyrrole can be used for the rational design of new bimetallic ORR catalysts.

## Conclusions

In this work, we synthesized a bioinspired bimetallic aerogel composed of manganese porphyrin and iron corrole (MnFe porphyrrole aerogel). To enhance its electronic conductivity, the aerogel underwent pyrolysis, allowing it to operate efficiently as both a substrate and a catalyst for the oxygen reduction reaction, thereby removing the necessity for additional carbon support. The resulting aerogel exhibited a three‐dimensional hierarchical structure, which can be further optimized to achieve an ideal balance between active site density and mass transport, featuring atomically dispersed manganese and iron centers. Notably, the coordination chemistry of the metal centers remained stable throughout the pyrolysis process.

The HT‐MnFe porphyrrole aerogel showcased remarkable catalytic performance, attaining an OCV of 0.97 V and a peak power density of 0.27 W cm^−2^ in an AEMFC, establishing its competitiveness within the landscape of electrochemical catalysts. Compared to the free base Fe porphyrrole, which exhibited a similar OCV of 0.97 V but suffered from a steeper voltage drop in the kinetic region and a lower peak power density of 0.20 W cm^−2^, the MnFe porphyrrole aerogel demonstrated significantly improved performance. In particular, the Mn‐containing aerogel exhibited a more stable voltage profile in the resistance‐limited region, indicating enhanced electronic conductivity and better mass transport properties. DFT calculations support these results by suggesting that the charge transfer from the porphyrin ring and the Mn ion to the Fe site is responsible for the enhanced ORR performance in the MnFe‐porphyrrole system.

These results emphasize the promising capabilities of the HT‐MnFe porphyrrole aerogel, along with the flexibility to manipulate the arrangement of metal centers, pore structures, and overall framework. Notably, this catalyst exhibits behavior that deviates from traditional scaling relations between O_2_‐intermediate binding energies, suggesting a disruption of the usual activity–selectivity trade‐offs that constrain conventional ORR systems. This deviation likely arises from the cooperative interactions between neighboring Mn and Fe centers within the heterometallic framework. Such advancements lay the groundwork for developing even more efficient and robust catalytic systems for ORR in fuel cells and a variety of other electrochemical processes in the future.

## Conflict of Interests

The authors declare no conflict of interest.

## Supporting information



Supporting information

## Data Availability

Data sharing is not applicable to this article as no new data were created or analyzed in this study.
