# Supplementary material for: Breaking the Linear Scaling Relations for the Oxygen Reduction Reaction with a Dual‐Atom Catalyst Composed of a MnFe‐Porphyrrole Aerogel
Source: Angew Chem Int Ed Engl. 2025 Sep 13;64(44):e202514013. doi: 10.1002/anie.202514013 (PMC12559461; doi:10.1002/anie.202514013)
Supplement: Supplementary file 1 — Supporting information [file ANIE-64-e202514013-s001.docx]

**Supporting information**

# Breaking the Linear Scaling Relations for the Oxygen Reduction Reaction with Dual-Atom Catalyst Composed of MnFe-Porphyrrole Aerogel

Eliana Lebowitz^1^, Prasenjit Das^1^, Łukasz Kielesiński^2^, Leigh Peles-Strahl^1^, David A. Cullen^3^, Ilya Grinberg^1^, Daniel T. Gryko*^2^, and Lior Elbaz*^1^

^1^ Chemistry Department, Bar-Ilan Center for Nanotechnology and Advanced Materials, Bar-Ilan University, Ramat-Gan 5290002, Israel

^2^ Institute of Organic Chemistry of Polish Academy of Sciences, Warsaw 01-224, Poland

^3^ Center for Nanophase Materials Sciences, Oak Ridge National Laboratory, Oak Ridge, TN 37831, USA

*Corresponding authors’ emails: dtgryko@icho.edu.pl; [lior.elbaz@biu.ac.il](https://poczta.onet.pl/NowaWiadomosc/Do/QlAkBFFKEBx2dgsdBXJ2cQQBfChCchE2Egk%2FZgEXARAfQkFWQwEGF1QnVkFWAwsCBAFWVkQTB3QWAFQfDRdDSQIKQwICQUdjJlBNGFIwai9IblhVZRQcVlEeZgRh)

Keywords: porphyrin, corrole, aerogel, oxygen reduction reaction, PGM-free, scaling relations

**Fe-corrole Synthesis**

**Scheme S1.** Synthesis of iron complex of corrole **2**.

**Synthesis of 5,10,15-tris(4-formylphenyl)corrolato iron(IV) chlorine complex (2).** Corrole **1** (0.090 g, 0.15 mmol) was dissolved in dry DMF (45 ml). Subsequently, anhydrous FeCl_2_ (0.381 g, 3.0 mmol) was added and the reaction mixture was stirred at 120 °C (oil bath) for 1 h under argon. Then, the mixture was cooled to room temperature and the solvent was evaporated. The precipitate was dissolved in DCM and extracted 7% solution of HCl. Organic layer was dried over Na_2_SO_4_, filtered and evaporated. The crude precipitate was purified by column chromatography (silica, DCM/MeOH 95:5). After column chromatography the mixture of two products (chloroiron(IV) corrole complex and *μ*-oxodiiron(IV) corrole complex) was observed, therefore the precipitate was disolved in DCM (100 ml) again, 7% solution of HCl (100 ml) was added and the mixture was stirred for two days at room temperature. After this time the mixture was separated and the organic layer was evaporated. Crude product was crystallized from DCM-hexane to afford product of analytical purity. It allowed to transform *μ*-oxodiiron(IV) corrole complex to the expected chloroiron(IV) corrole complex.

**Characterization**

The ^1^H NMR spectrum was recorded using a Varian 600 spectrometers. Chemical shift (δ ppm) was determined with TMS as the internal reference, *J* values are given in Hz. High resolution mass spectrum (HRMS) was obtained by atmospheric pressure chemical ionization (APCI). Chromatography was performed on silica gel 60 (230-400 mesh) and thin layer chromatography was performed on TLC plates (Merck, silica gel 60 F_254_).

**5,10,15-tris(4-formylphenyl)corrolato iron(IV) chlorine complex (2).** Red-brown precipitate (0.083 g, Yield 79%) Mp 245 °C (decomp). ^1^H NMR (600 MHz, CDCl_3_): δ 23.98 (bs, 2H; *β*-H), 22.77 (bs, 2H; *β*-H), 21.90 (bs, 2H; *β*-H), 10.83 (s, 2H; CHO), 10.57 (s, 1H; CHO), 5.54 (bs, 2H; *β*-H or Ar), -1.83 (s, 2H; Ar), -2.06 (s, 2H; Ar), -2.81 (s, 1H; Ar), -2.93 (s, 1H; Ar), -5.93 (bs, 2H, Ar), -6.73 (bs, 2H, Ar), -38.84 (bs, 2H, *β*-H or Ar). HRMS (APCI) *m/z*: calcd for C_40_H_23_ClFeN_4_O_3_, 698.0808 [M]^-^; found, 698.0807.

**Figure S1.** ^1^H-NMR spectra of corrole **2.**

**Scheme S2.** Synthesis of porphyrrole gel.

**Koutecky-Levich**

**Figure S2.** Koutecky-Levich graph based on RRDE measurements taken at 225, 400, 625, 900, and 1225 rpm at 0.80 V vs. RHE.

**DFT calculations**


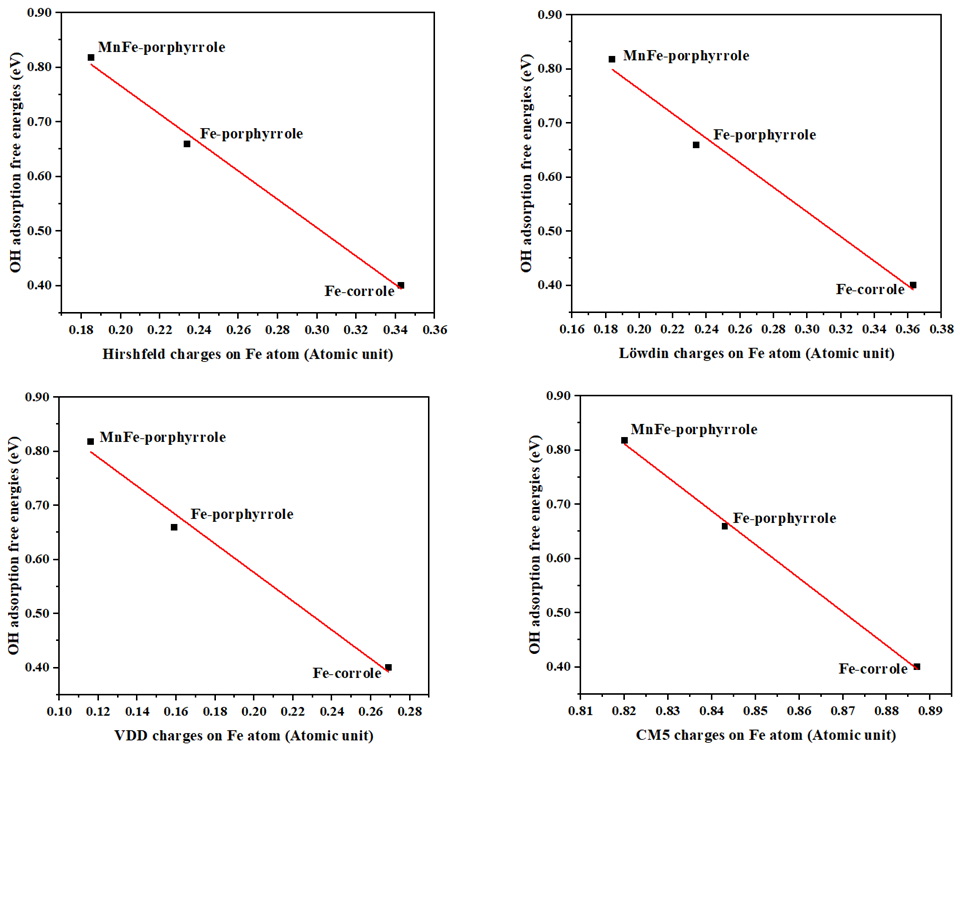


**Figure S3.** Correlations between the Hirshfeld, Löwdin, VDD, and CM5 charges on the Fe atom and the DFT-calculated values of OH adsorption free energies (Δ*G*_ads,OH_).

Experimental Methods

Aerogel synthesis: The aerogels were synthesized through a condensation reaction involving a porphyrin with amino substituents and a corrole containing aldehyde constituents. 4.36 mg of manganese porphyrin, specifically Mn(III) 5,10,15,20-tetra-4-aminophenyl porphyrin (PorphyChem) was dissolved in 250 μL of DMSO. In a separate vial, 5.33 mg of Fe(II) 5,10,15-tris(4-formylphenyl) corrole (preparation procedure described in SI) was dissolved in 250 μL of DMSO. This solution was then combined with the porphyrin solution and agitated for thirty seconds. The resultant solution was maintained at 80 °C overnight to allow the reaction to progress and for gel formation. Once the gel was formed, it was washed with DMSO to eliminate any unreacted reagents until the wash solvent above the gel was colorless. Subsequently, the gel was washed with acetone to replace the solvent within the gel, preparing it for supercritical drying. Supercritical CO_2_ drying was performed using a critical point dryer (Leica EM CPD300 Auto). After obtaining the aerogel, a pyrolysis process was conducted in a glass-tube oven (Thermo Scientific-Lindberg Blue M) under a continuous argon flow (150 sccm). The temperature was increased from 25°C to 200°C over 20 minutes and held at 200°C for 2 hours. The temperature was then raised from 200°C to 800°C at a rate of 2°C/min, maintaining this temperature for an additional two hours. The chosen pyrolysis conditions were based on prior research.^1^

Material Characterization: ICP to analyze the metal content was conducted using SPECTRO ARCOS ICP-OES, multi view, FHX22. For ICP analysis, 2.01 mg of the sample was pyrolyzed in air at 500 °C for 20 hours, then dissolved in aqua regia, followed by dilution with DIW. X-ray photoelectron spectroscopy (XPS) was performed with Thermo Scientific Nexsa system, while HAADF-STEM imaging was carried out using Nion UltraSTEM 100-aberration corrected STEM. The instrument operated at 60kV with a probe semiconvergence of approximately 31mrad. BET surface area measurements were taken using N_2_ adsorption isotherms at 77 K with a Quantachrome Autosorb iQ instrument. The surface area was calculated based on the BET model, and the pore size distribution was determined using DFT models.

Electrochemical Measurements: RRDE experiments were conducted using Bio-Logic VMP 300 bipotentiostat. 2 mg of the pyrolyzed aerogel were finely ground and mixed in a solution of DIW and isopropanol in a 1:4 volume ratio, along with 0.4% Nafion, to form a 10 mg/mL slurry. The slurry was sonicated for one hour before use. 10 µL of slurry were applied to the glassy carbon disk on the RRDE electrode (Pine), followed by drying at room temperature. The catalyst loading on the electrode was 0.4 mg ^-2^. The measurements were performed in a three-electrode setup (Pine), with the RRDE serving as the working electrode, a glassy carbon rod as the counter electrode, and a reversible hydrogen electrode (RHE) as the reference. All experiments were carried out in an alkaline solution of 0.1 M KOH (Sigma-Aldrich) prepared with DIW. The ORR onset potential was defined as the potential at which a current density of 100 mA cm^-2^ was achieved, and the limiting current was measured at 0.5 V vs. RHE.

Fuel Cell Measurement: The HT-MnFe porphyrrole aerogel was evaluated in an anion exchange membrane fuel cell (AEMFC). For the cathode, a slurry was prepared by grinding the aerogel powder and mixing it with DIW and isopropanol in a 1:2 volume ratio. An ionomer solution (PiperION Anion Exchange Dispersion, 5 wt%) was then added until the ionomer-to-catalyst weight ratio reached 3:7. The slurry underwent sonication for one hour in an ice bath, followed by overnight stirring and a second round of sonication. The anode slurry was prepared using PtRu/C (Pt 40%, Ru 20% on carbon black, HiSPEC 10000, Alfa Aesar), mixed with ionomer solution and a DIW/isopropanol solution in the same ratios as the cathode slurry. The slurry was also sonicated for one hour in an ice bath, stirred overnight, and sonicated again. Both slurries were sprayed on a 1 cm^2^ BC-29 substrate using a SonoTek ultrasonic spray system. The anode catalyst loading was 2.0 mg cm^-2^ of PtRu, while the cathode loading was 1.8 mg cm^-2^ of the MnFe aerogel. For activation, both GDEs and the membrane (PiperION-A-20-HCO3) were soaked in 0.5 M NaOH for one hour while the solution was exchanged every twenty minutes. The MEA was then assembled without hot pressing and tested using an 850e Scribner Associates fuel cell test station. The cell was operated at 80 °C, with hydrogen supplied at a flow rate of 150 sccm and a humidifier temperature of 77 °C and a relative humidity (RH) of 88.5%. Oxygen was delivered to the cathode with a humidifier temperature of 77 °C as well, with an RH of 88.5% and a flow rate of 500 sccm. For the break-in procedure, the cell was held at 0.02 A for a half hour until the potential stabilized. Following this, an I-V curve was recorded. Measuring the current at constant voltage beginning at OCV and sequentially decreasing to 0.78 in 0.04 V intervals, followed by 0.06 V intervals for the remainder of the measurement. At each voltage step, the cell was held for 120 s to ensure stabilization, and current data was collected at a rate of one measurement per second. The 120 recorded current values at each voltage step were averaged to obtain one point at each voltage step for the I-V plot.

DFT calculations: We optimized the geometries of the model systems and the intermediates using the Perdew–Burke–Ernzerhof (PBE) functional and def2-SVP basis set for all atoms.^2-4^ We used this functional because the OH adsorption energies obtained with this functional in previous studies for metal-corrole and metal-porphyrin systems show good agreement with results from more sophisticated computational approaches and experimental onset potentials.^5,6^ To confirm that the optimized structures correspond to local minima on the potential energy surface, we performed frequency calculations with the same functional and basis set. Furthermore, we refined the energies of intermediates by single-point energy calculations using the def2-TZVP and def2-TZVPP basis sets using the optimized geometries obtained by calculations with the def2-SVP basis set.^4^ The Gaussian 16 program was utilized for all calculations in this work.^7^

**References**

1 Zion, N. *et al.* Porphyrin aerogel catalysts for oxygen reduction reaction in anion‐exchange membrane fuel cells. *Advanced Functional Materials* **31**, 2100963 (2021).

2 Perdew, J. P., Burke, K. & Ernzerhof, M. Generalized gradient approximation made simple. *Physical review letters* **77**, 3865 (1996).

3 Perdew, J. P., Ernzerhof, M. & Burke, K. Rationale for mixing exact exchange with density functional approximations. *The Journal of chemical physics* **105**, 9982-9985 (1996).

4 Weigend, F. & Ahlrichs, R. Balanced basis sets of split valence, triple zeta valence and quadruple zeta valence quality for H to Rn: Design and assessment of accuracy. *Physical Chemistry Chemical Physics* **7**, 3297-3305 (2005).

5 Nagaprasad Reddy, S., Krishnamurthy, C. B. & Grinberg, I. First-principles study of the ligand substituent effect on ORR catalysis by metallocorroles. *The Journal of Physical Chemistry C* **124**, 11275-11283 (2020).

6 Samala, N. R. & Grinberg, I. Tuning of ORR activity through the stabilization of the adsorbates by hydrogen bonding with substituent groups. *Physical Chemistry Chemical Physics* **22**, 27811-27817 (2020).

7 Frisch, M. e. *et al.* (Gaussian, Inc. Wallingford, CT, 2016).
